# Hartmann’s procedure vs loop colostomy in the treatment of obstructive rectosigmoid cancer

**DOI:** 10.1186/1749-7922-9-52

**Published:** 2014-10-04

**Authors:** Slobodan Krstic, Vladimir Resanovic, Tamara Alempijevic, Aleksandar Resanovic, Ana Sijacki, Vladimir Djukic, Zlatibor Loncar, Aleksandar Karamarkovic

**Affiliations:** University of Belgrade, Belgrade, Serbia; Clinic for Emergency Surgery, Emergency center, Clinical centre of Serbia, Belgrade, Serbia; Clinic for Gastroenterology, Clinical Center of Serbia, Belgrade, Serbia; Surgery Department, Medical Center “Bezanijska kosa”, Belgrade, Serbia

**Keywords:** Hartmann’s procedure, Loop colostomy, Obstructive rectosigmoid cancer, ASA score

## Abstract

**Introduction:**

Colorectal carcinoma is the most common malignant gastrointestinal tumour. There is still a considerable controversy when it comes to urgent surgical treatment of obstructive carcinoma of the left colon and rectum.

**Methods:**

Seventy-five patients from the randomized trial were followed up. This study was designed as a stratified randomized trial with four stratums according to age and ASA score (older/younger than 60 years and ASA score <>3). Each of the four groups is then divided into two sub-groups according to the operating technique: loop colostomy or Hartmann’s procedure.

**Results:**

There were no difference found in hospitalization among the groups (loop colostomy vs. Hartmann’s procedure) in the same stratus (P = 0.3192, P = 0.5760, P = 0.9023 respectively), except in the case of doing reconstructive procedure after loop colostomy (P = 0.0049) in the fourth stratum (patients younger than 60 years with ASA score lower than 3). Type of operation had no influence over the blood test values observed on admittance and during hospitalization (P = 0.319, P = 0.871, P = 0.7, P = 0.843, P = 0.52 respectively for the blood values). In terms of surgical and non-surgical complications it has been shown that there is no statistically significant difference between patients treated by two methods. Age, gender, ASA score, type of operation and surgical complications were not singled out as a risk factor for fatal outcome (P = 0.199, P = 0.155, P = 0.764, P = 0.452 and P = 0.724 respectively). The only factors that are singled out as a risk factor for death are the emergence of non-surgical complications and angina pectoris (P = 0.006, P = 0.001).

**Conclusions:**

There is no difference in surgical treatment of large bowel obstruction caused by rectosigmoid carcinoma. Neither of those two methods showed significant advantage in treatment of large bowel obstruction caused by rectosigmoid cancer.

## Introduction

Colorectal carcinoma is the most common malignant gastrointestinal tumour. Intestinal obstruction is an acute surgical condition. It is believed that about 60% of mechanical bowel obstruction is caused by colorectal tumours, 20% by diverticulosis and about 5% of intestinal obstruction is caused by a colonic volvulus. Despite the significant progress made in the field of screening, prevention and early diagnosis of colorectal cancer, it is known that 20% of patients with these tumours as the first symptom have signs of intestinal obstruction [1–4]. Intestinal obstruction leads towards impaired respiratory function due to reduced diaphragmatic excursions, while the intraluminal microbial proliferation increases the risk of infection [2, 3]. Emergency surgery is therefore associated with significant morbidity and mortality in these conditions, which ends, in most cases, by creating a colostomy (temporary or permanent) [1, 2, 5, 6].

There is still a considerable controversy when it comes to urgent surgical treatment of obstructive carcinoma of the left colon and rectum. To resolve this clinical entity there are several possibilities: loop colostomy and subsequent resection (in two or three stages), resection with end colostomy (Hartmann’s procedure), and resection and primary anastomosis [7]. The focus of this paper will be on loop colostomy and Hartmann’s procedure.

The Association of Coloproctology of Great Britain and Ireland have found that there are four important predictors of outcome: age, ASA score, the need for emergency surgery and Dukes classification [5]. In a poll conducted by the Society of American Gastrointestinal and Endoscopic Surgeons, 67% of surgeons opted for Hartmann’s procedure in high-risk patients and 26% of them for loop colostomy [8]. Emergency surgery was identified as an independent risk factor of mortality after surgery, according to the French Association of Surgery investigation. Based on these few studies (only one of them was designed as randomized), there is a recommendation (Consensus conference of the World Society of Emergency Surgery (WSES) and peritoneum and surgery (PnS) society) that Hartmann’s procedure should be preferred to loop colostomy (level B recommendation II) [9].

We aimed to evaluate both of these procedures and to show which one is more adequate in case of emergency surgery, using stratified randomized trial. Non-surgical complications and angina pectoris emerged as risk factors for poor outcome, while there was no difference in terms of surgical complications for both surgical procedures.

## Material and methods

In this study, 75 patients with obstructive malignant lesion of rectosigmoid carcinoma were included and operated by five experienced surgeons. Patients who had a malignant peritoneal dissemination and patients who were unable to cooperate or who were in poor general health were excluded. Age, gender, hospital stay, surgical and non-surgical complications and blood transfusions (intraoperative and postoperative) were recorded. The aim of the study was to compare two emergency surgical procedures (Hartmann’s procedure vs. loop colostomy) used in cases of acute mechanical obstruction caused by rectosigmoid cancer. This study was designed as a stratified randomized trial with four stratums (groups) according to age and ASA score (older/younger than 60 years and ASA score <>3). Each of the four groups is then divided into two sub-groups according to the operating technique: loop colostomy or Hartmann’s procedure.

A loop colostomy is mostly performed for creation of a temporary stoma to divert stool away from an area of intestine that has been blocked due to cancer. This surgery brings a loop of bowel through an incision in the abdominal wall. The loop is held in place outside the abdomen by a plastic rod slipped beneath it. An incision is made in the bowel to allow the passage of stool through the loop colostomy. The supporting rod is removed approximately seven to ten days after surgery, when healing has occurred that will prevent the loop of bowel from retracting into the abdomen.

Hartmann’s operation or Hartmann’s procedure is the surgical resection of the rectosigmoid colon with closure of the rectal stump and formation of an end colostomy. During this procedure, the lesion is removed, the distal bowel closed intraperitoneally and the proximal bowel diverted with a stoma. High ligation of limfo vascular pedicle wasn’t mandatory for surgeon due to possible significant bowel distension, although it was performed whenever possible.

Statistical analysis was performed with SPSS 18.0 program. Apart from descriptive statistic methods (mean, standard deviation), we used *t*-test and chi-squared test for quantitative comparisons. P < 0.05 was considered statistically significant.

The study was approved by Ethics Committee of the Clinical Center of Serbia, written informed consent was obtained from each human subject and the patients had been operated and postoperatively closely monitored in Emergency center of the Clinical centre of Serbia.

## Results

Our study group consisted of 36 men and 39 women, while the mean age was 66.31 ± 12.50 (27 – 99) years. Mean duration of hospitalization in our study group was 10.92 ± 6.85 days (2–35 days). There were no difference found among the groups (loop colostomy vs. Hartmann’s procedure) in the same stratus (P = 0.3192, P = 0.5760, P = 0.9023 respectively). The only significant statistic difference in hospitalization was found in the case of doing reconstructive procedure after loop colostomy (P = 0.0049) in the fourth stratum (patients younger than 60 years with ASA score lower than 3).

Type of operation had no influence over the blood test values observed on admittance and during hospitalization (P = 0.319, P = 0.871, P = 0.7, P = 0.843, P = 0.52 respectively for the blood values). In terms of surgical and non-surgical complications it has been shown that there is no statistically significant difference between patients treated by loop colostomy and Hartmann’s procedure (Table 1). The amount of transfused blood was without any statistical difference in each of four stratums (P = 0.689, P = 0.848, P = 0.495 P = 0.687 respectively). In terms of intraoperative transfusion, we showed that there wasn’t any statistical difference (P = 0.303, P = 0.0557, P = 0.272, P = 0.7183 respectively). The difference in overall mortality for patients operated by these two techniques was not statistically significant (P = 0.45).

**Table 1:** **Observed characteristics in the function of type of operation**

Observed characteristics		Type of operation		
		Loop colostomy	Hartmann’s procedure	
Age (X ± SD)		65.68 ± 14.828	66.93 ± 11.020	^b^p = 0.678
Gender	m	12 (42.90%)	23 (50.00%)	^a^p = 0.551
	f	16 (57.10%)	23 (50.00%)	
Randomization groups	> 60 god, ASA ≥ 3	12 (16.20%)	17 (23.00%)	^a^p = 0.265
	> 60 god, ASA < 3	4 (5.4%)	16 (21.60%)	
	< 60 god, ASA ≥ 3	3 (4.10%)	3 (4.10%)	
	< 60 god, ASA < 3	9 (12.20%)	10 (13.50%)	
Surgical complications	Yes	2 (2.7%)	2 (2.7%)	^a^p = 0.579
	No	25 (34.2%)	44 (60.3%)	
Nonsurgical complications	Yes	3 (4.1%)	9 (12.3%)	^a^p = 0.347
	No	24 (32.9%)	37 (50.7%)	

Age, gender, ASA score, type of operation and surgical complications were not singled out as a risk factor for fatal outcome (P = 0.199, P = 0.155, P = 0.764, P = 0.452 and P = 0.724 respectively, Table 2). The only factors that are singled out as a risk factor for death are the emergence of non-surgical complications and angina pectoris (P = 0.006, P = 0.001). Relative risk for fatal outcome is 1.49 for patients with non-surgical complications and 4 for patients with angina pectoris. Blood transfusion, chronic renal failure and diabetes mellitus have no impact on survival rate (P = 0.427, P = 0.285 and P = 0.81 respectively).

**Table 2:** **Risk factors for fatal outcome**

Observed risk factor	P value
Age	P = 0.199
Gender	P = 0.155
ASA score	P = 0.764
Type of operation	P = 0.452
Surgical complications	P = 0.724
Non-surgical complications	P = 0.006
Angina pectoris	P = 0.001

**Figure 1:**
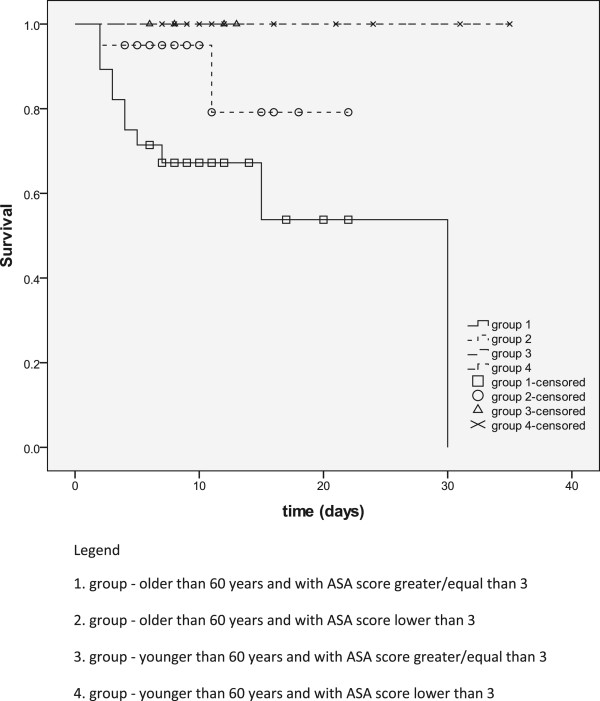
**Intrahospital survival according to randomization groups.**

Using the Kaplan-Meier method, we have shown that statistically significant difference exists only between two randomization groups (Figure 1) in terms of intrahospital mortality: older than 60 years with ASA > 3 and younger than 60 years with ASA <3 (P = 0.001).

## Discussion

Colorectal cancer is the third most common cancer in the United States, with similar incidence of both men and women. Also, it is the second cause of cancer deaths. Moreover, 53% of all colorectal cancers occur in sygmoid colon and rectum [10]. Recent studies showed that there is an increasing incidence of rectosigmoid and rectal cancer in patients younger than 40 years [11–13].

Colon cancer may be detected in patients with symptoms or may show as a result of screening programme. Despite great progress made in colorectal cancer [CRC} screening, at least 20% of CRC cases is diagnosed on surgical operation due to large bowel obstruction [1–4].

In our study we observed patients presented with large bowel obstruction due to rectosigmoid cancer with no previous data on CRC. Mean duration of hospitalization in our study was 10.92 ± 6.85 days. In the terms of hospitalization, we have showed that there was no significant difference among these groups, except in stratum 4. This difference can be explained by the fact that randomized patients in this group were younger than 60 years and their ASA score was lower than 3. In this stratum (young, healthy and working population) patients underwent reconstructive surgical intervention during the same hospitalization. These patients were operated in a shortest possible time interval because they are relatively young and working population. In this manner, we have tried to minimize recovery period after surgical interventions and to speed up return to work. Comparing Hartmann’s procedure (63 patients) with loop colostomy (58 patients), Kronborg showed in his randomized trial that there was significant difference in hospital stay which was shorter in the group treated with Hartmann’s procedure [14].

Type of operation had no statistical significant influence on blood test values during the hospitalization. In other words, both surgical procedures had similar blood loss and had no significant impact on the amount of transfused blood. In addition, the rate of surgical and nonsurgical complications showed no statistically significant difference between patients treated by these two techniques. Therefore it can be said that both surgical techniques (loop colostomy and Hartman’s technique) are equally good and adequate methods of resolving intestinal obstruction.

In terms of mortality, there weren’t any statistical significant difference among all groups, except in the case of older patients with ASA greater than 3. These data are in perfect match with study performed by Kronborg, which has been the only known randomized study so far with similar scientific focus. The data obtained in our paper are also in full compliance with Cochrane database review done by DeSalvo et al. [15].

Age, gender, ASA score, type of operation and surgical complications were not singled out as a risk factor for fatal outcome. Non-surgical complications and angina pectoris were the only risk factor for poor outcome. Non-surgical complications are thought primarily to complications from cardiovascular system, which are more common in case of elderly patients with higher values of ASA score.

Using Kaplan Meier analysis of intrahospital mortality, we showed that in only one case there was a highly statistically significant difference between the two randomization groups. It is a group of subjects older than 60 years with ASA score greater than 3 and a group of patients younger than 60 years with ASA score of less than 3. It was the first group where the rate of non-surgical complication was more common, as evidenced by the proven fact that these complications are predictors of poor outcome. Poor outcome is obviously associated with cardiovascular diseases and non-surgical complications.

The results of this study show that there is no difference in surgical treatment of large bowel obstruction caused by rectosigmoid carcinoma. Both methods, loop colostomy and Hartmann’s procedure, had similar impact on mortality and hospitalization. Neither of those two methods showed significant advantage in treatment of large bowel obstruction caused by rectosigmoid cancer (mortality and morbidity). The blood loss is similar in both surgical procedures, according to the need for transfusion (intra and postoperative).

Colonic resection and primary anastomosis is a third surgical approach for resolving acute intestinal obstruction caused by rectosigmoid cancer. In the multicentric study by Kube et al. [16] conducted in Germany, 743 patients underwent emergency surgery for obstructive rectosigmoid carcinoma, performed as radical resection. Resection with primary anastomosis has been done in 57.9% cases, resection with anastomosis and protective stoma in 11.7% and Hartmann’s procedure in 30.4% patients. In the last group patients were multimorbid, overweight and male. There was no statistical significant diference among those groups regarding morbidity and intrahospital mortality. Also, protective stoma didn’t improve the rate of anastomotic leakage. The results of this study showed that Hartmann’s procedure should be done in high-risk patients and advanced obstruction. Meyer [17] in his review concluded that Hartmann’s procedure remains relevant in high-risk patients operated during the night and weekend, when the most experienced surgeons aren’t always available. Furthermore, Jung et al. [18] showed that patients older than 75 years were more likely to undergo Hartmann’s procedure even in the case of rectal tumors without colonic obstruction.

According to our findings, Hartmann’s procedure is recommended only in older, high risk patients (high ASA score), with advanced and neglected rectosigmoid obstructive tumors and expressed proximal bowel distention. In our study group, 68% of patients were older than 60 years, and most of them presented with neglected rectosygmoid tumors, followed by bowel distention and endangered colon vitality. These patients, with numerous comorbidities, can not be subjected to primary anastomosis after resection, so they underwent Hartmann’s procedure and, in addition, high ligation of limfo vascular pedicle.

In the group of patients treated with Hartmann’s procedure, we had two patients with wound infection. In the group of patients treated with loop colostomy, two patients had also wound infection as surgical complications. There was no rectal stump dehiscence/leak and no abscess formation and wound dehiscence. As mentioned before, there is no significant difference regarding the rate of surgical complications. When it comes to the non-surgical complications, we showed that they occure more frequently, but again, there is no significant difference according to the type of operation. Myocardial decompensation was the most frequent (50%), followed by pneumonia (33%) and anxiety (17%). Emergence of non-surgical complications singled out as one of the risk factors for poor outcome.

According to recent research, a combination of endoscopy and laparoscopy is an appropriate therapeutic strategy. Placing a preoperative self-expanding metallic stents (SEMS) allows bowel movements and subsequent laparoscopic surgery. Preoperative self-expanding metallic stents insertion does not adversely affect oncological outcomes and patient survival [19]. This approach guarantees a minimum invasiveness, without compromising the effectiveness of treatment. Watt et al. [20] compared the rate of complications after SEMS insertion and emergency surgery for obstructive left colon cancer, while Zhang et al. [21] analyzed 6 retrospective studies and 2 randomized controlled trials (meta analysis) concerning combined endoscopic and surgical approach versus surgery alone. According to their results, there was no statistical significance for permanent rate of colostomy and postoperative mortality (30 days follow-up), although they were lower in SEMS group. Zhao et al. [22] in their report analyzed five randomized controlled trials concerning semielective surgery after SEMS and emergency surgery. They showed that SEMS insertion followed by semielective surgery for obstructive rectosigmoid carcinoma compared to emergency surgery had decreased the rate of postoperative complications, surgical site infections (SSI) and had enhanced the rate of primary anastomosis, which had decreased the rate of colostomy. They didn’t detect any statistical significance considering rate of anastomotic leak, primary anastomotic rate and 30 days postoperative mortality.

According to these researches, it can be concluded that SEMS insertion is very promising method that facilitates surgery and provides better oncologic results. However, Liu et al. [23] did the meta-analysis of complications of colonic stenting versus emergency surgery for acute left-sided malignant colonic obstruction. They analyzed Medline, Embase and the Cochrane library in order to evaluate the complications of these procedures. There was no significant difference in hospital death and complications between those two approaches. Unfortunately, this method is still unavailable in our country due to the unfavorable economic situation, but we would like to compare this modern method to older ones.

## Conclusion

It can be concluded that Hartmann’s procedure (with additional high ligation of limfo vascular pedicle) is better suited to older people because in this manner the tumour is removed, which is quite good if patient can’t be ready for next reconstructive operation in a shorter period of time. Loop colostomy could be advised for younger, healthy patients that can be ready for immediate definitive surgery (tumor removal) in a shortest possible period, i.e. two or three weeks. In this case, surgeon on the second operation has better comfort and less bowel inflation facilitates better oncological approach (mandatory high ligation of limfo vascular pedicle) which eventually would very likely improve surgical and oncological results.

## Abbreviations

ASA: American Society of Anaestesiologists
CRC: Colorectal cancer
SEMS: Self expanding metallic stents
SSI: Surgical site of infection
GDP: Gross domestic product.
